# Adaptation and qualitative evaluation of encounter decision aids in breast cancer care

**DOI:** 10.1007/s00404-018-5035-7

**Published:** 2019-01-16

**Authors:** Pola Hahlweg, Isabell Witzel, Volkmar Müller, Glyn Elwyn, Marie-Anne Durand, Isabelle Scholl

**Affiliations:** 1 Department of Medical Psychology, University Medical Center Hamburg-Eppendorf, Martinistraße 52, 20246 Hamburg, Germany; 2 Department of Gynecology, University Medical Center Hamburg-Eppendorf, Martinistraße 52, 20246 Hamburg, Germany; 3 The Dartmouth Institute for Health Policy and Clinical Practice, Dartmouth College, Level 5, Williamson Translational Research Building, One Medical Center Drive, Lebanon, NH 03756 USA

**Keywords:** Breast cancer, Shared decision-making, Encounter decision aids, Option Grid™ decision aids, Cross-cultural adaptation

## Abstract

**Purpose:**

Shared decision-making is currently not widely implemented in breast cancer care. Encounter decision aids support shared decision-making by helping patients and physicians compare treatment options. So far, little was known about adaptation needs for translated encounter decision aids, and encounter decision aids for breast cancer treatments were not available in Germany. This study aimed to adapt and evaluate the implementation of two encounter decision aids on breast cancer treatments in routine care.

**Methods:**

We conducted a multi-phase qualitative study: (1) translation of two breast cancer Option Grid™ decision aids; comparison to national clinical standards; cognitive interviews to test patients’ understanding; (2) focus groups to assess acceptability; (3) testing in routine care using participant observation. Data were analysed using qualitative content analysis.

**Results:**

Physicians and patients reacted positively to the idea of encounter decision aids, and reported being interested in using them; patients were most receptive. Several adaptation cycles were necessary. Uncertainty about feasibility of using encounter decision aids in clinical settings was the main physician-reported barrier. During real-world testing (*N* = 77 encounters), physicians used encounter decision aids in one-third of potentially relevant encounters. However, they did not use the encounter decision aids to stimulate dialogue, which is contrary to their original scope and purpose.

**Conclusions:**

The idea of using encounter decision aids was welcomed, but more by patients than by physicians. Adaptation was a complex process and required resources. Clinicians did not follow suggested strategies for using encounter decision aids. Our study indicates that production of encounter decision aids alone will not lead to successful implementation, and has to be accompanied by training of health care providers.

**Electronic supplementary material:**

The online version of this article (10.1007/s00404-018-5035-7) contains supplementary material, which is available to authorized users.

## Introduction

In breast cancer treatment, patients and physicians have to make several decisions regarding treatment options that might considerably affect patients’ quality of life and other person-centred health outcomes. With many new emerging treatment options, this process has become more complex and challenging for patients and physicians over the last years. It is, therefore, important to take patients’ preferences and values into account [1, 2]. Strategies to support these challenging decision-making processes are needed. Studies showed that the majority of patients wants to be well informed and participate in decision-making processes [3–7].

Patient participation can be achieved through shared decision-making (SDM). SDM is an interactional process between patient and physician [1]. The aim is for both parties to be actively involved and come to a shared and informed treatment decision based on the available clinical evidence and the patient’s individual preferences and values [8]. Within this process, the physician supports the patient to weigh the benefits and risks as well as possible consequences of different treatment options [9]. Since patients and physicians appraise the quality of different treatment options differently [10], it is important to encourage a dialogue and come to a shared understanding of what the best option for the individual patient is.

Routine cancer care often does not meet patients’ preferences optimally [11, 12]. Decision aids (DAs) are supporting materials that facilitate the involvement of patients in decision-making processes by depicting different treatment options with their respective benefits and risks [13]. Many DAs (e.g. brochures or videos) contain detailed information for patients to read before or after the clinical encounter. Recently, short DAs (so-called encounter decision aids, EDAs), which can be used during the clinical encounter, were developed and evaluated [14, 15]. EDAs have shown to support the implementation of SDM [16–18]. A Cochrane review of 105 studies involving a total of 31,043 participants showed positive effects of DAs on several outcomes, e.g. DAs improved the correct perception of benefits and risks of different options, reduced decisional conflict, and increased active involvement of patients [14]. However, the length of many DAs hinders the implementation in routine practice [19]. Short EDAs to support SDM during the clinical encounter seem more promising for changing the dialogue [20, 21]. EDAs are currently lacking in many languages (e.g. German) [22], and little is known about needs for cross-cultural adaptation of translated EDAs.

Thus, this study aimed to adapt and pilot test the translations of two EDAs for breast cancer treatment: (a) surgical options for breast cancer and (b) options for breast reconstruction after mastectomy. This included (1) the translation and cross-cultural adaptation of the EDAs for breast cancer treatment, (2) the evaluation of the patients’ and physicians’ acceptance of the translated EDAs, and (3) the evaluation of the EDAs’ feasibility in routine care.

## Methods

### Study design

We conducted a qualitative cross-sectional study. In phase 1 we translated and adapted two English EDAs into German. Pilot testing included an assessment of acceptance (phase 2), and testing in the real-world setting (phase 3). We followed the COREQ reporting guideline (cp. supplementary material) [23].

### Option Grid™ DAs

The EDAs used in this study are Option Grid DAs. Those are one-page documents consisting of a table filled with the most important information on different treatment options. Each line in the tables stands for one frequently asked question (FAQ) regarding the topic. In the columns, the answers for each option are given (cp. supplementary material). Physicians can use those tables during the clinical encounter to explain the different treatment options, facilitate comparison, and come to a shared decision. The Option Grid Collaborative, a consortium of researchers, clinicians, and patient representatives from the US and Great Britain, developed a multitude of such Option Grid DAs [24, 25].

### Setting and subjects

We cooperated with a certified comprehensive breast cancer centre at a university hospital in Germany. Inclusion criteria for patients participating in phases 1 and 2 (interviews and focus groups) were as follows: (1) diagnosed with breast cancer and not currently facing decisions about surgery or breast reconstruction, (2) age 18 years and older. Exclusion criteria were insufficient knowledge of German language or severe cognitive impairment. The inclusion criterion for physicians participating in phase 2 (focus groups and interviews) was a specialization in breast cancer care. Phase 3 (participant observations) took place at the breast centre during consultation hours at the outpatient clinic.

### Recruitment

Prior to participation, we informed participants about the study and obtained written informed consent.

We used purposive sampling aiming to include participants with diverse demographic characteristics in phases 1 and 2, convenience sampling in phase 3. We recruited patients for interviews and focus groups face-to-face in collaboration with staff at the breast centre and with a patient organization. Physicians participating in focus groups and interviews were recruited through collaboration partners and email inquiries to various breast cancer facilities in the metropolitan region of Hamburg. We offered participants in phases 1 and 2 a compensation fee of 25 Euros. Patients participating during participant observations were recruited face-to-face at the breast centre by study team members.

### Data collection

Participants in phases 1 and 2 completed a short demographic survey.

#### Phase 1: translation and adaptation

The translation procedure was derived from the TRAPD protocol [26, 27]. Necessary adaptations to German clinical standards were discussed with breast cancer specialists in our multidisciplinary team (IW, VM). National guidelines [28] and current evidence-based developments were taken into account. Patient comprehensibility was tested in cognitive interviews at the university hospital (conducted by PH, female psychologist experienced in interviewing) with *N* = 9 patients [29]. Cognitive interviews followed a guideline developed within this study, lasted about 1 h, and were audio-recorded. After adaptations, the final translated versions of the Option Grid DAs were mailed to *N* = 10 patients accompanied by a short survey asking if the adaptation led to improvements, and if final versions were well understandable.

#### Phase 2: assessment of acceptance

Final German versions of the Option Grid DAs were tested, based on the recommendations of the International Patient Decision Aids Standards (IPDAS) Collaboration, which developed internationally accepted standards for DAs [30].

First, we tested acceptance of the EDAs through focus groups and interviews with patients and physicians specialized in breast cancer (phase 2, [30]). The planned sample size was 16–20 for both physicians and patients or until data saturation. Focus groups lasted 120 min, took place at the university hospital, followed a guideline developed within the study and were chaired by IS and PH, two female psychologists experienced in administering focus groups. We offered physicians and patients, unable to participate in a focus group, individual interviews instead (conducted by PH). Focus groups and interviews were audio-recorded.

#### Phase 3: testing in the real-world setting

Prior to participant observations in the real-world setting (phase 3, [30]), physicians working at the breast centre received a 60-min group training in administering the EDAs. This training consisted of general information on SDM and EDAs, a guideline for the use of Option Grid DAs, and an example video. Physicians were asked to use the Option Grid DAs when consulting with patients facing one of the relevant treatment decisions. Feasibility of using the German Option Grid DAs was assessed at the breast centre over a period of 4 weeks. During the first week, we sought to observe as many clinical encounters as possible to gain an overview; for the following 3 weeks, we pre-selected potentially relevant encounters in collaboration with staff at the breast centre to save resources. One researcher (PH, female psychologist experienced in participant observation [31, 32]) carried out the observations. We recorded observations on a pre-structured form (cp. supplementary material). The observer elaborated barriers for the use of the EDAs either by explicitly asking the physicians, or by drawing conclusions about potential barriers from the observed situation.

### Data analysis

First, one person (PH) cumulated all comments and suggestions from the audio-recordings of the cognitive interviews into one document. Second, the relevance of all comments and suggestions was discussed in the study group (IS, PH). Taking into account the physician feedback (given by IW, VM) and the results of the cognitive interviews with patients, final German versions of the two EDAs were established after consultation with the developers (GE, MAD).

Audio-recordings were transcribed verbatim; field notes of participant observations were digitalized. Transcripts were not returned to participants for approval. We analysed the anonymized qualitative data following the principles of Mayring’s qualitative content analysis, a systematic, rule-guided approach to analyse text by categorizing relevant themes and sub-themes [33]. Two members of the research team (PH, IS) carried out the analyses using MAXQDA software (version 10, VERBI GmbH, Berlin, Germany).

## Results

### Phase 1: translation and adaptation

The translation of the Option Grid DAs with two translators (PH; WF, cp. acknowledgements) and one reviewer (IS) was feasible. The adaptation process was more extensive than expected, because several feedback circles between the core executive study team, breast care specialists, and developers at the Option Grid collaborative were necessary to establish agreement on the pre-final German versions. Physicians voiced the necessity to include new clinical evidence into the DAs (e.g. regarding survival rates after breast conserving therapy or mastectomy [34]), and make minor additions in content. It was also necessary to adapt, so the content would match the current state of breast cancer care delivery in Germany.

Compare Table 1 for demographic characteristics of patients participating in cognitive interviews. Cognitive interviews with patients showed overall good understanding of the Option Grid DAs. Only a few changes in the DAs were made to reflect the results of the cognitive interviews and additional results from focus group discussions (e.g. wording of certain medical terms, order of FAQs, and clarification of minor logical incongruities). Again, the core executive study team and the developers at the Option Grid collaborative discussed and agreed on the final German versions.

**Table 1:** Demographic and clinical characteristics of patients in phases 1 and 2

	Phase 1: cognitive interviews with patients (*N* = 9)		Phase 2: focus groups and interviews with patients (*N* = 13^a^)	
	Mean (SD)	Range	Mean (SD)	Range
**Age** (in years)	53.3 (11.0)	32–66	57.2 (11.1)	30–71

	*N*	%	*N*	%
**Sex**: Female	9	100	13	100
**Mother tongue**				
German	8	89	12	92
Other	1	11	0	0
Missing	0	0	1	8
**Level of education**				
Low^b^	2	22	0	0
Intermediate^c^	3	33	5	38
High^d^	4	44	8	62
**Surgical procedure**				
Lumpectomy	3	33	9^e^	69^e^
Mastectomy	5	56	6^e^	46^e^
Missing	1	11		
Reconstruction	3	33	3	23
**Additional treatments**				
Chemotherapy	6	67	9	69
Radiation	6	67	8	62
Anti-hormonal therapy	4	44	4	31

*N* sample size, *SD* standard deviation, *BCT* breast conserving therapy

^a^Adequate data saturation reached

^b^Years of education completed ≤ 9

^c^Years of education completed 10–12

^d^Years of education completed ≥ 13

^e^Two cases indicated lumpectomy and mastectomy

Eight of ten patients, who received the final versions of the DAs by mail, responded. They indicated that the EDAs were well (*N* = 4) to very well (*N* = 4) understandable. Seven of the eight patients indicated improvement of the EDAs during the adaptation process.

Compare supplementary material for the final German versions of the Option Grid DAs.

### Phase 2: acceptance of the German Option Grid DAs

Acceptance was assessed in a sample of *N* = 13 patients (two focus groups, three interviews) and *N* = 13 physicians (one focus group, seven interviews). Tables 1 and 2 show demographic characteristics of patients and physicians.

**Table 2:** Demographic characteristics of breast cancer specialists in phase 2

Phase 2: focus groups and interviews with physicians (*N* = 13^a^)		
	Mean (SD)	Range
**Age** (in years)	46.4 (10.5)	31–60
**Work experience** (in years)	17.4 (9.4)	4–35

	*N*	%
**Sex**: Female	7	54
**Work setting**		
Breast centre	8	62
Private practice	5	38

*N* sample size, *SD* standard deviation

^a^Adequate data saturation reached

Physicians and especially patients valued the idea of Option Grid DAs and expressed interest in using them. They thought of the EDAs as supporting tools for patients and physicians during and after the clinical encounter. One physician said, “In my opinion it [the EDA] is clearly structured and I think it is very helpful for patients; especially that they can look at it afterwards [i.e. after the clinical encounter]”. A patient described how she felt during initial diagnosis as follows, “I could not concentrate. I could not absorb any information. I thought I had understood everything very quickly. At home everything had vanished […] and that is why I think this slip of paper [the EDA] is good”. Some physicians and few patients were more sceptical regarding the EDAs. One physician said, “I think that one can also cause information overload for patients with this sheet”.

On the one hand, most participants thought that Option Grid DAs are probably not suitable for all patients, because of, e.g. diverse personality traits and cognitive abilities. On the other hand, several participants indicated that all patients should be offered the DAs. Physicians emphasized that they do not feel comfortable with handing out EDAs to patients prior to a first face-to-face encounter.

We found six categories of influencing factors for the acceptance of the Option Grid DAs, which are described in Table 3.

**Table 3:** Factors influencing the acceptance of the Option Grid DAs

Influencing factors	Description
**Factors increasing acceptance**	
Helpful during the clinical encounter	The EDAs were thought to stimulate questions, and support the clinical encounter
Helpful after the clinical encounter	Patients could take the EDAs home
**Factors with mixed feedback**	
Factors regarding the EDA itself	This included the information on the EDAs, the structure of the EDAs, linguistic aspects, and the balance between offering detailed information and being short
Emotional aspects	Especially patients emphasized that the DAs could reduce anxiety. Some physicians voiced that the DAs could unsettle and overburden patients
**Factors decreasing acceptance**	
Factors regarding feasibility	This included prerequisites for using the EDAs (e.g. EDAs need to be embedded in clinical encounter and not stand alone; use of the EDA should be introduced as an offer not a must do). Structural barriers were mentioned (e.g. time pressure, one encounter including communication of the diagnosis and treatment decision). The right point in time for the administration of the EDAs was controversially discussed
Questioning the preference sensitivity of the decisions depicted in the two EDAs	Physicians questioned the nature of the decisions as preference-sensitive

### Phase 3: feasibility assessment

Eighty-nine of 103 invited patients (86.4%) consented to participate. Over a period of 4 weeks, we observed clinical encounters of 66 of those 89 distinct patients (74.2%). In 11 of the observed cases, we additionally observed a follow-up visit scheduled within the 4-week-timeframe. This led to *N* = 77 clinical encounters included in the analyses. Visits lasted between 3 and 65 min (mean 18.1, SD 11.9). In 29 of the 77 encounters (37.7%), one or more significant others of the patient were present. Most often this was a partner/spouse (15 out of 77 visits, 19.5%), friend (7 out of 77 visits, 9.1%), parent (4 out of 77 visits, 5.2%), or grown-up child (4 out of 77 visits, 5.2%). For descriptive statistics on the current diagnoses, see Table 4.

**Table 4:** Current state of disease

Disease	Frequency	%
Primary breast cancer	29	37.7
Metastatic breast cancer	15	19.5
Suspected breast cancer	12	15.6
DCIS	6	7.8
Recurrent breast cancer	4	5.2
With a history of breast cancer	4	5.2
Other^a^	7	9.1

^a^E.g. genetic mutation, cyst of the breast, micro-calcifications

Thirty-three (42.9%) of the 77 clinical encounters focused on surgery planning (breast surgery and/or reconstruction). In twelve of those 33 potentially relevant visits (36.4%), physicians broached the issue of the Option Grid DAs. In nine of those twelve visits, one or both of the EDAs were used (twice both EDAs, five times surgical options EDA, twice breast reconstruction EDA). In the other three of those twelve encounters, physicians referred to the EDAs used in the prior clinical encounter, but did not look at the EDAs again. After three additional encounters, where physicians did not broach the issue of Option Grid DAs with the patients, physicians mentioned to the observer that using the tool would have been useful with that patient.

If Option Grid DAs were used, physicians explained the format shortly. A detailed elaboration of different options on the EDA was not observed. Patients only had little time to familiarize themselves with the EDAs during the clinical encounters. In one encounter, a family-member read the EDA during the patient’s physical examination and asked the physician questions afterwards. Apart from this encounter, patients only briefly looked at the EDA during the encounter or did not look at it at all. In seven of the nine cases, the patient took the EDA with her after the clinical encounter.

Barriers to the use of EDAs were clinical reasons (e.g. physician did not consider decision preference-sensitive), structural aspects (e.g. lack of time), and individual patient characteristics (e.g. language barriers). Facilitators for the use of EDAs were (a) decision-making process being spread out over more than one clinical encounter, (b) patient giving positive feedback about the EDAs and therewith reinforcing the use, and (c) patient being overwhelmed by only verbal information.

## Discussion

Our results suggest that physicians and patients value the idea of EDAs and show interest in using them during and after clinical encounters. However, we needed several cycles of adaptation to reach adequate acceptance by German physicians and patients. Several physicians questioned the feasibility of using EDAs in breast cancer care. Testing in routine care showed that physicians used Option Grid DAs in one-third of potentially relevant encounters. However, if Option Grid DAs were used, they were not discussed in depth during the clinical encounter.

This is the first study of its kind and therewith adds important insight for following research on EDAs. The good acceptance we found for the Option Grid DAs provides a basis for additional research on short EDAs. However, this is a pilot study. The sample size suffices for this kind of study (i.e. qualitative study aiming to gain insight into attitudes and processes). This lays ground for larger studies assessing effectiveness and feasibility of EDAs to produce results with better generalizability in the future. We cannot appraise within this study, how much the presence of the observer motivated physicians to use the EDAs. Also, if patients engaged in reading the EDA after the clinical encounter was not assessed within this study.

This study emphasizes the importance of thorough adaptation of translated DAs. We found that the discussion of discrepancies with different stakeholders of the target population and the developers of the original EDAs was a fruitful albeit time-intensive process. To pay attention to those adaptation needs is the first step towards generating a well-accepted tool.

The main challenges for the use of Option Grid DAs were doubts about the feasibility of the EDAs in routine cancer care. Other studies also found that medical staff appraises the incorporation of DAs in existing clinical routines as challenging [35, 36] and that DAs are not sufficiently implemented in routine care [19].

A current study on the use of Option Grid DAs for several treatment decisions showed that physicians ask for more training and feedback on the use of Option Grid DAs [20]. In our study, physicians did not discuss the options on the DAs during the clinical encounter. Possibly, our short physician training was not sufficient to enable physicians to elaborate on the EDAs during the encounter. More training and feedback might have strengthened the use of the tools. This is in line with implementation science that suggests that behaviour change is best achieved through a combination of initial training and ongoing support [37].

The next step in research and practice could be to assess methods to support the implementation of the available adapted EDAs. This would be especially valuable since new studies showed that the use of EDAs supports the implementation of SDM [16, 17]. Barriers reported in this study such as uncertainty about the right moment to administer the DAs, time pressure, or the questioning of preference-sensitivity need to be addressed in training and support efforts. Besides practical and structural barriers, physicians’ attitude towards SDM is essential for EDAs to be successful.

At the same time, DAs are only one way to support SDM. Légaré and colleagues emphasized that we need interventions that target different aspects of the decision-making process to successfully foster the implementation of SDM in routine care [38]. It is extremely important that implementation programs are multi-faceted and based on implementation science. The cluster randomized implementation study that is currently conducted at the University Medical Center Hamburg-Eppendorf is one example of such a multi-faceted implementation program [39].

## Conclusion

This study provides German versions of two EDAs for breast cancer treatment that have been thoroughly adapted with attention to cross-cultural factors. It was shown that after adaptation acceptance is promising overall. However, the implementation of EDAs needs to be facilitated. The next step will be to find ways to implement EDAs in routine breast cancer care, and therewith support the implementation of SDM.

## Electronic supplementary material

Below is the link to the electronic supplementary material.

Supplementary material 1: Original English version of the Option Grid DA "Breast cancer: surgical options" (PDF 975 kb)

Supplementary material 2: Original English version of the Option Grid DA "Breast reconstruction after surgery for cancer: options" (PDF 992 kb)

Supplementary material 3: Field note form (PDF 125 kb)

Supplementary material 4: German version of the Option Grid DA "Breast cancer: surgical options" (PDF 702 kb)

Supplementary material 5: German version of the Option Grid DA "Breast reconstruction after surgery for cancer: options" (PDF 696 kb)

Supplementary material 6: COREQ checklist (PDF 1081 kb)

## Data Availability

The dataset supporting the conclusions of this article is available upon request for researchers after consultation with the corresponding author and the responsible Ethics Committee. Please contact the corresponding author, Pola Hahlweg (Email: p.hahlweg@uke.de), if you wish to request the data set.

## Abbreviations

BCT: Breast conserving therapy
DA: Decision aid
EDA: Encounter decision aid
FAQ: Frequently asked questions
GE: Glyn Elwyn
IPDAS: International Patient Decision Aids Standards
IS: Isabelle Scholl
IW: Isabelle Witzel
MAD: Marie-Anne Durand
*N*: Sample size
PH: Pola Hahlweg
SD: Standard deviation
SDM: Shared decision-making
VM: Volkmar Müller
WF: Wiebke Frerichs
