# Supplementary material for: Adaptation and qualitative evaluation of encounter decision aids in breast cancer care
Source: Arch Gynecol Obstet. 2019 Jan 16;299(4):1141–9. doi: 10.1007/s00404-018-5035-7 (PMC6435605; doi:10.1007/s00404-018-5035-7)
Supplement: Supplementary file 3 — Supplementary material 3: Field note form (PDF 125 kb) [file 404_2018_5035_MOESM3_ESM.pdf]

Hahlweg, P., Witzel, I., Müller, V., Elwyn, G., Durand, M.-A., Scholl, I.

Adaptation and Qualitative Evaluation of Encounter Decision Aids in Breast Cancer Care.

Corresponding author: Pola Hahlweg, p.hahlweg@uke.de

Additional file 3. Field note form.

Field notes – participant observation

**observer:**	**date:**	**time of observation:**	
**persons involved** (tick appropriate and specify if applicable):			
☐ patient	☐ physician<br>(☐ senior / ☐ junior)	☐ nursing staff	☐ family member / friend
☐ other persons, please specify:			
**diagnosis:**	**reason for encounter**:		

<u>Observation memo:</u>

Option Grid DA used? yes ☐ no ☐ / ☐ breast cancer surgery
☐ breast reconstruction

„Explain it“:

„Give it“:

„Use it“:

Option Grid DA given to patient to take it home? yes ☐ no ☐

Observable patient reaction to Option Grid DA:

Additional observations and impressions:
