# Supplementary material for: Adaptation and qualitative evaluation of encounter decision aids in breast cancer care
Source: Arch Gynecol Obstet. 2019 Jan 16;299(4):1141–9. doi: 10.1007/s00404-018-5035-7 (PMC6435605; doi:10.1007/s00404-018-5035-7)
Supplement: Supplementary file 4 — Supplementary material 4: German version of the Option Grid DA "Breast cancer: surgical options" (PDF 702 kb) [file 404_2018_5035_MOESM4_ESM.pdf]

Hahlweg, P., Witzel, I., Müller, V., Elwyn, G., Durand, M.-A., Scholl, I.
Adaptation and Qualitative Evaluation of Encounter Decision Aids in Breast Cancer Care in Germany.
Corresponding author: Pola Hahlweg, p.hahlweg@uke.de
Additional file 4. German version of the Option Grid DA "Breast cancer: surgical options".

Brustkrebsoperation

Sie können diese Übersicht als Hilfestellung benutzen, um mit Ihrem Arzt/Ihrer Ärztin darüber zu sprechen, wie Brustkrebs behandelt werden kann.

Häufig gestellte Fragen	Brusterhaltende Operation mit Strahlentherapie	Brustentfernung (medizinischer Begriff: Mastektomie)
**Was wird bei der Operation entfernt?**	Der Tumor und etwas umliegendes Gewebe (Sicherheitsabstand) werden entfernt.	Die gesamte betroffene Brust wird entfernt.
**Wie hoch ist die Langzeit-überlebensrate?**	Die Überlebensrate ist bei beiden Behandlungsmöglichkeiten gleich.	
**Wie hoch ist die Wahrscheinlichkeit, dass der Krebs in der betroffenen Brust bzw. Brustwand zurückkommt?**	Innerhalb von 10 Jahren nach der brusterhaltenden Operation oder der Brustentfernung kommt der Brustkrebs bei etwa 5 bis 10 von 100 Frauen (5 bis 10%) zurück. Das Risiko ist abhängig vom Stadium des jetzigen Krebses und den Eigenschaften des Tumors. Bitte besprechen Sie dies mit Ihrem Arzt.	
**Müssen Lymphknoten in der Achselhöhle entfernt werden?**	Wenn Krebszellen in den Lymphknoten in der Achselhöhle gefunden werden, wird Ihr Arzt / Ihre Ärztin mit Ihnen weitere Behandlungen besprechen. Dies kann zum Beispiel eine Operation der Achselhöhle und/oder Strahlentherapie der Lymphknoten in der Achselhöhle sein.	
**Brauche ich mehr als eine Operation?**	Möglicherweise. Bis zu 20 von 100 Frauen (20%) benötigen eine weitere Operation, um weiteres von Krebs befallenes Gewebe oder Lymphknoten zu entfernen.	Ja, wenn Sie sich für einen späteren Brustwiederaufbau entscheiden. Möglicherweise, um Lymphknoten zu entfernen.
**Wie lange brauche ich, um mich von der Operation zu erholen?**	Die meisten Frauen können innerhalb von 2-3 Tagen nach der Operation entlassen werden. Ihre gewohnten Aktivitäten werden Sie nach etwa ein bis zwei Woche (manchmal länger) wieder ausführen können.	Die meisten Frauen können innerhalb von 3-5 Tagen nach der Operation entlassen werden. Ihre gewohnten Aktivitäten werden Sie nach mehreren Wochen (manchmal länger) wieder ausführen können.
**Brauche ich Strahlentherapie?**	Ja. Sobald Ihre Wunden ausreichend verheilt sind, werden Sie etwa fünfmal die Woche Strahlentherapie erhalten. Die Strahlentherapie dauert etwa 6 Wochen.	Möglicherweise brauchen Sie auch nach der Brustentfernung Strahlentherapie.
**Brauche ich Chemotherapie?**	Möglicherweise. Dies ist abhängig vom Stadium des Krebses und den Eigenschaften des Tumors, aber unabhängig davon, ob Sie eine brusterhaltende Operation oder Brustentfernung erhalten.	
**Werden mir die Haare ausfallen?**	Chemotherapie führt meistens zu Haarausfall. Bestrahlung der Brust oder Achselhöhle führt nicht zu Haarausfall.	

Sie können die Rückseite des Option Grids nutzen, um sich Notizen zu machen oder Fragen aufzuschreiben.

Autoren (deutsche Version): Isabelle Scholl, Pola Hahlweg, Isabell Witzel, Volkmar Müller
Deutsche Version vom: 01.10.2015; *gültig bis:* 01.10.2017
Autoren (engl. Version): Glyn Elwyn, Lisa Caldon, Kari Rosenkranz, Dale Collins Vidal, Marie-Anne Durand, Stephanie Sivell, Malcolm Reed
Zugrundeliegende wissenschaftliche Studien (englisch): http://www.optiongrid.org/resources/breastcancer_evidence.pdf
Website mit der aktuellsten englischen Version: http://www.optiongrid.org
