# Supplementary material for: Adaptation and qualitative evaluation of encounter decision aids in breast cancer care
Source: Arch Gynecol Obstet. 2019 Jan 16;299(4):1141–9. doi: 10.1007/s00404-018-5035-7 (PMC6435605; doi:10.1007/s00404-018-5035-7)
Supplement: Supplementary file 5 — Supplementary material 5: German version of the Option Grid DA "Breast reconstruction after surgery for cancer: options" (PDF 696 kb) [file 404_2018_5035_MOESM5_ESM.pdf]

Hahlweg, P., Witzel, I., Müller, V., Elwyn, G., Durand, M.-A., Scholl, I. Adaptation and Qualitative Evaluation of Encounter Decision Aids in Breast Cancer Care in Germany.
Additional file 5. German version of the Option Grid DA "Breast reconstruction after surgery for cancer: options".

option grid®

Operation zum Brustwiederaufbau (Brustrekonstruktion)

Sie können diese Übersicht als Hilfestellung benutzen, um mit Ihrem Arzt / Ihrer Ärztin darüber zu sprechen, ob Sie nach der Brustentfernung einen Brustwiederaufbau wünschen.

Häufig gestellte Fragen	Kein Brustwiederaufbau (nur Brustentfernung)	Sofortiger Brustwiederaufbau	Späterer Brustwiederaufbau
**Was wird während der Operation gemacht?**	Die gesamte betroffene Brust wird entfernt. Dabei bleibt eine flache Brustwand zurück. Sie werden eine Prothese erhalten, die sie als Einlage in Ihrem BH tragen können.	In einer Operation wird sowohl die gesamte Brust entfernt als auch eine neue Brust aufgebaut. Dafür wird entweder ein Implantat oder körpereigenes Gewebe verwendet.	In einer ersten Operation wird die gesamte Brust entfernt. Dabei bleibt eine flache Brustwand zurück. In einer weiteren Operation wird zu einem späteren Zeitpunkt eine neue Brust aufgebaut.
**Wird es durch den Brustwiederaufbau schwieriger festzustellen, ob der Krebs zurückgekommen ist?**	Trifft nicht zu, da kein Brustwiederaufbau.	Nein. Durch regelmäßige Untersuchungen der wiederaufgebauten Brust können Veränderungen erkannt werden.	
**Wie hoch ist die Wahrscheinlichkeit, dass der Krebs in der betroffenen Brust bzw. Brustwand zurückkommt?**	Innerhalb von 10 Jahren kommt der Brustkrebs trotz der Brustentfernung bei etwa 5 bis 10 von 100 Frauen (5 bis 10%) zurück.		
**Was sind die häufigsten Probleme nach der Operation?**	Langsame Wundheilung, starke Narbenbildung, Hauteinziehungen, Taubheitsgefühl, Nachblutungen, eingeschränkte Beweglichkeit.	Langsame Wundheilung, starke Narbenbildung, Hauteinziehungen, Taubheitsgefühl, Nachblutungen, eingeschränkte Beweglichkeit. Bis zu 20 von 100 Frauen (20%) sind nicht zufrieden mit Größe und Form der neuen Brust und brauchen eine weitere Operation, um beide Brüste ähnlicher zu machen.	
**Bei Brustwiederaufbau mit körpereigenem Gewebe: Was passiert an der Körperstelle, wo das Gewebe entnommen wird?**	Trifft nicht zu, da kein Brustwiederaufbau.	An der Körperstelle, wo das Gewebe entnommen wurde, wird eine Narbe zurückbleiben. Es ist selten, dass hierdurch eine Muskelschwäche entsteht, die Ihre gewohnten Aktivitäten beeinträchtigt.	
**Werden weitere Behandlungen (z.B. Chemotherapie, Strahlentherapie) durch den Brustwiederaufbau verzögert?**	Trifft nicht zu, da kein Brustwiederaufbau.	Möglicherweise, da Ihre Wunden zuerst verheilen müssen. Dies bedeutet nicht, dass die Krebsbehandlungen weniger wirksam sind.	Nein. Sie erhalten die weiteren Krebsbehandlungen vor dem Brustwiederaufbau.
**Wie lange dauert es, bis ich meine gewohnten Aktivitäten wieder ausführen kann?**	Etwa 4 Wochen (ohne Chemotherapie und/oder Strahlentherapie).	Etwa 3 bis 6 Monate nach dem Brustwiederaufbau. Dies ist abhängig von der Art des Brustwiederaufbaus (Implantat oder Eigengewebe).	
**Wird die Brustwarze weg sein?**	Ja.	Möglicherweise kann die Brustwarze erhalten bleiben oder es kann später eine neue Brustwarze aufgebaut werden.	Eine neue Brustwarze kann später aufgebaut werden.
**Wie wird sich die neue Brust anfühlen?**	Etwa 10 von 100 Frauen (10%) empfinden ein gewisses Spannungs- oder Straffheitsgefühl in der Brustregion.	In der wiederaufgebauten Brust wird sich das Tastgefühl verändern. Dennoch sagen 70 von 100 Frauen (70%), dass sich die neue Brust wie ein Teil des eigenen Körpers anfühlt.	In der wiederaufgebauten Brust wird sich das Tastgefühl verändern. Dennoch sagen 80 von 100 Frauen (80%), dass sich die neue Brust wie ein Teil des eigenen Körpers anfühlt.

Sie können die Rückseite des Option Grids nutzen, um sich Notizen zu machen oder Fragen aufzuschreiben.

Autoren (deutsche Version): Isabelle Scholl, Pola Hahlweg, Isabell Witzel, Volkmar Müller*; Deutsche Version vom:* 1.10.2015; *gültig bis:* 1.10.2017
Autoren (engl. Version): Glyn Elwyn, Lisa Caldon, Kari Rosenkranz, Dale Collins Vidal, Marie-Anne Durand, Stephanie Sivell, Malcolm Reed
Zugrundeliegende wissenschaftliche Studien (englisch): http://www.optiongrid.org/resources/breastcancer_evidence.pdf
Website mit der aktuellsten englischen Version: http://www.optiongrid.org
